# Genetic Heterogeneity Reveals On-Going Speciation and Cryptic Taxonomic Diversity of Stream-Dwelling Gudgeons (Teleostei, Cyprinidae) in the Middle Danubian Hydrosystem (Hungary)

**DOI:** 10.1371/journal.pone.0097278

**Published:** 2014-05-13

**Authors:** Péter Takács, Péter Bihari, Tibor Erős, András Specziár, Ildikó Szivák, Péter Bíró, Eszter Csoma

**Affiliations:** 1 Department of Hydrozoology, Balaton Limnological Institute, MTA Centre for Ecological Research, Tihany, Hungary; 2 Institute of Genetics, MTA Biological Research Centre, Szeged, Hungary; 3 Department of Medical Microbiology, University of Debrecen, Debrecen, Hungary; Smithsonian Conservation Biology Institute, United States of America

## Abstract

Although stream-dwelling gudgeons (Cyprinidae, genus: *Gobio*) are widespread in Central Europe, the taxonomy of this group and the distribution of its species are still unexplored in detail. The aims of our study are to ascertain taxonomic composition and distribution of the former *Gobio gobio* superspecies in the inner area of the Carpathian Basin. Since the presence of cryptic species is suspected in this area, we examined the taxonomic and phylogenetic relationships of Central European *Gobio* taxa by sequencing the mitochondrial DNA control region (mtCR). Additionally, we characterized the genetic structure of 27 stream-dwelling gudgeon populations of this area by Amplified Fragment Length Polymorphism (AFLP). Results of mtCR analysis proved the presence of three species already known as *G. obtusirostris* (dominant in NW-Hungary), *G. gobio* (sporadic) and *G. carpathicus* (sporadic). Additionally, the analysis revealed the existence of one doubtful taxon, *G. sp1* (dominant in NE-Hungary), and a new isolated haplogroup (dominant in SW-Hungary). Although Network analysis showed significant detachment among haplogroups, their genetic distances were quite small. Therefore Bayesian phylogenetic analysis showed weak nodal support for the branching pattern both for newly described haplotypes, and for the already accepted species. AFLP data showed distinct population structure and a clear pattern of isolation was revealed by distance of stocks. At the same time, level of separation was not affected by the altitudinal position of sites. Moreover we found three major clusters of populations which were separated according to hydrographic regions, and corresponded to the findings of mtCR analysis. Our results suggest the on-going speciation of gudgeons in the Carpathian Basin, however the separation of haplogroups seems to only be an intermediate phase. The discovered natural pattern seems to be only slightly influenced by anthropogenic impacts. Additionally our results put into question the suitability of the recently accepted within *Gobio* genus taxonomy.

## Introduction

Freshwaters are exceedingly diverse ecosystems, but at the same time they are extremely sensitive to habitat degradation and pollution [1]. Accurately quantifying their taxonomic and functional diversity is a fundamental requirement of conservation biological, ecological, biogeographical, and macroevolutionary research [2], [3], [4]. However, when species are not clearly distinguishable by the conventional methods using ecological and morphological traits, have highly similar environmental needs and reveal a high level of phenotypic plasticity, then estimates of biodiversity and ecosystem functioning will be biased [5], [6], [7], [8].

With the increasing use of molecular techniques, it has become evident that many species formerly believed to have widespread geographical distribution can in fact be divided into numerous more or less discrete entities -so called cryptic or sibling species by Mayr [9]- or represent genetic gradients between separating species (i.e. on-going speciation) [10], [11], [12], [13], [14]. Such phenomena are more likely to occur in organisms with limited dispersion ability and/or in organisms living in separated or narrowly connected habitats [15], [16], [17]. Many stream-dwelling fishes have specific environmental needs and therefore form discrete populations, not only between geographical areas with separated catchment systems, but also between closely related sites of the same catchment [18], [19], [20], [21]. These isolated fish populations may then genetically differentiate with time, although they may still maintain their similar morphological appearance and ecological function [22]. However, this process is not yet fully understood in seemingly well connected catchment systems.

The type species of the *Gobioninae* subfamily (Fam: Cyprinidae), the common gudgeon *Gobio gobio* Linnaeus (1758), was known as the most widely distributed lentic gudgeon species in West Eurasia [23]. However, the high between and within population morphological variability of this taxon [24] resulted a long-standing debate regarding its taxonomical status [25], [26]. As a result, numerous forms/varietas/subspecies were described, nearly all from larger catchment areas within its range (e.g. [23]). Recent genetic studies have raised some of the former subspecies to species level and several new species have been described as well [27], [28] [29], [30], [31], [32]. However, the taxonomic and genetic status of gudgeon populations in the Carpathian Basin is still unclear. *Gobio gobio* was considered a common species in the waters of the Carpathian Basin for a long time [33], [34]. On the contrary, recent studies [31], [35] excluded the Carpathian Basin from the potential range of *G. gobio* and suggested the occurrence of the Danube gudgeon, *Gobio obtusirostris* Valenciennes (1842) in the western region of the basin, and the Carpathian gudgeon, *Gobio carpathicus* Vladykov (1925) in the drainage system of River Tisza. Furthermore, Mendel et al. [31] indicated the presence of a “species” (sic!: *Gobio sp1*) from the catchment of River Tisza, but the taxonomic position of this newly described taxon has not yet been firmly established. It is also important to note that the findings of Kottelat and Freyhof [35] are based exclusively on data from the literature. Moreover, although the study of Mendel et al. [31] is the most comprehensive genetic study on the Middle European *Gobio* species to date, it included only a very limited number of samples from the Carpathian Basin and furthermore all of those samples originated from the edges of this region. The above mentioned *Gobio* taxa (i.e. *G. gobio, G. obtusirostris, G. carpathicus* and *G. sp1*) are morphologically very similar [35], [36] and thus their distribution and ecology cannot be explored without molecular justification [24]. Furthermore, recent studies on *G. gobio* and the related species [37], [38], [39], [40] found remarkably high genetic and morphologic variability between and within catchment areas, supporting the likelihood of the presence of cryptic species.

This study aims to ascertain taxonomic composition and distribution of the former *G. gobio* superspecies in the inner Carpathian Basin. Specifically, we (i) examine the taxonomic and phylogenetic relationships of *Gobio* taxa and the presence of cryptic species, and (ii) characterize the genetic structure of gudgeon populations with special attention paid to the effects of hydrological distance and elevation as possible forces facilitating genetic separation.

To unravel the taxonomic relationships of stream-dwelling gudgeons inhabiting the central area of the Carpathian Basin we use the same methodology and molecular marker (sequencing the mitochondrial Control region) as was used by Mendel et al. [31], thus our results are comparable with their findings. Moreover, we also screened for Amplified Fragment Length Polymorphisms (AFLP’s) as a supplementary method of analysis for genome-wide genetic variation [41].

## Materials and Methods

### Ethics Statement

This study was carried out following relevant national and international guidelines pertaining to the care and welfare of fish. Collections were made by electrofishing, partially from sampling sites which are located within protected areas. Moreover, each species within the *Gobioninae* subfamily is protected in Hungary. Electrofishing in protected areas and any procedure (collection and storage of tissue samples) to be applied to protected species are subject to authorisation in Hungary. Fin tissue collection and storage were approved by the National Inspectorate for Environment, Nature and Water, Hungary (permission numbers: 14/3714-2/2009, 14/1237/2/2010, 14/881/5/2011, 14/678-9/2012). Fish collected for this study were narcotized using clove oil. After fin tissue sampling, when they regained consciousness, they were returned to the wild. Field studies did not involve fish that were endangered (The IUCN Red List of Threatened Species v. 2013.1; www.iucnredlist.org).

### Study Area

**Figure 1:**
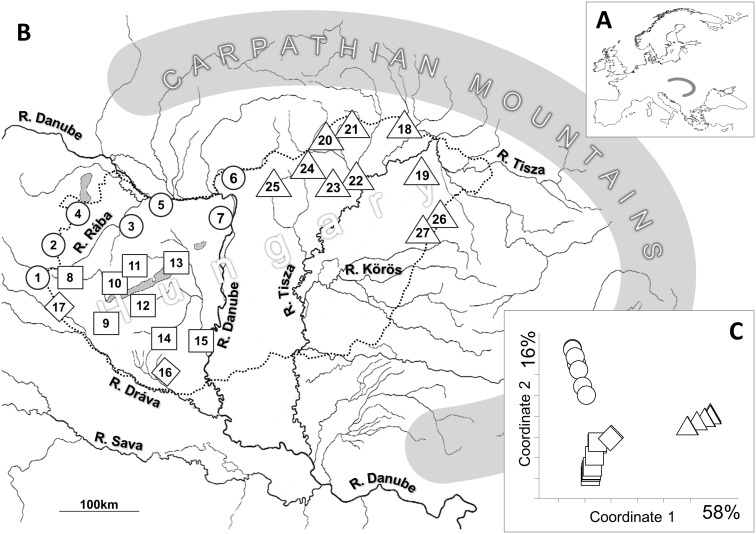
Location of the Carpathian Basin in Europe (A), location of the sampling sites (1–27) in the Carpathian Basin (B) and PCoA representation of hydrologic distances between the sampling sites (C). Hungarian country border is marked with the dotted line. Different symbols refer to sites belonging to different catchment areas: ○ - North Danubian; □ - Middle Danubian; ⋄ - South Danubian and ▵ - the Tisza River catchment. For detailed information see Table 1.

**Figure 2:**
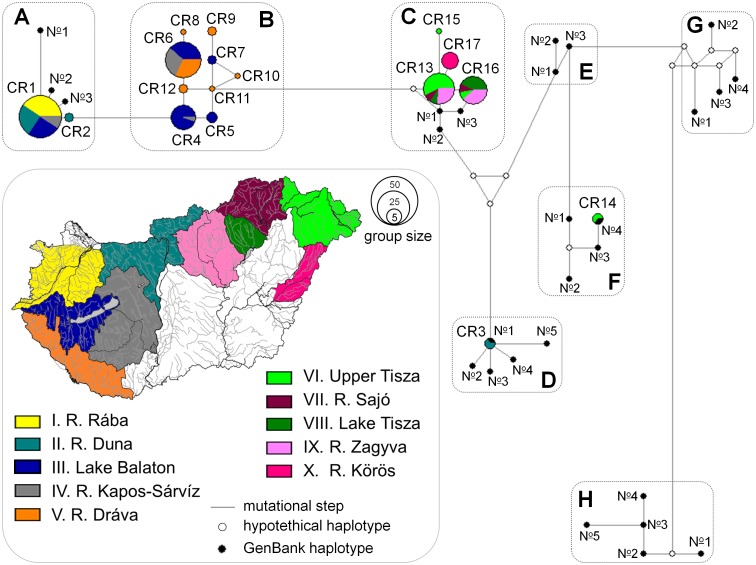
Median-Joining network of mtCR sequence data relating *Gobio spp.* with previously published data. Circle size is relative to the number of individuals carrying the same haplotype. Line length refers to the genetic distances of haplotypes. Small open circles represent median vectors (missing or theoretical haplotypes). CR01–17: Haplotypes of the 168 specimens analysed in this study. Letter code of haplogroups/“boxes” and numbers (N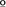 ) of previously published haplotypes in each box correspond with the numbers and codes displayed in Table 2.

All sampling sites were situated in the inner area of the Carpathian Basin, which belongs to the drainage system of the Danube River. Based on its topographic characteristics, the Hungarian part of Middle Danubian hydrosystem can be divided into two larger catchments and ten smaller sub-catchments (Table 1, Figures 1 and 2). The hydrography of this area is biaxial. From the western region, all watercourses empty into the Danube River. The eastern part of the country belongs to the drainage system of the Tisza River, which is the largest tributary of the Danube (157 000 km^2^ catchment area). The structure of this drainage system is dendritic, with the Tisza River forming the central axis. All the studied watercourses connected to the middle section of the River Tisza [42], therefore this region was not differentiated further. The hydrography of the Danubian system is more complicated. This system consists of three comparatively isolated subsystems: North, Middle and South Danubian regions (Table 1). North Danubian region is formed by the drainages of River Rába (Raab), River Ipoly (Ipel) and by the drainages of some direct inflowing streams. Middle Danubian catchment originally joined to the River Danube through a marshy area. Until the construction of the Sió canal at the end of the 19th century, there was only intermittent connection between the Danube and the Lake Balaton drainage system. Therefore this subdrainage had been hydrologically isolated to some degree from the others until the last century. Waters from South Danubian region flow into the River Dráva (Drau), which empties to the Danube River at Osijek (Croatia).

**Table 1:** Name, location, code, geographic position, and altitude (alt.) of sampling sites, the number of individuals (N) analysed by the two methods (mtCR, AFLP).

N 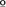	Drainage	Region	Sub-catchment area	Name	Code	Latitude	Longitude	alt.(m)	mtCR (N)	AFLP (N)
1.	Danubian	NorthDanubian	I. River Rába	Szölnöki stream	Sz	N46.92730	E16.20149	235	2	10
2.				River Pinka	Pi	N47.15667	E16.46333	222	3	9
3.				Gerence stream	Ge	N47.35146	E17.61271	178	3	10
4.				River Répce	Re	N47.44407	E16.67898	194	10	10
5.			II. “direct inflows”of River Danube	Cuhai-Bakony-érstream	Cu	N47.65584	E17.88013	121	10	10
6.				Kemence stream	Km	N47.99175	E18.91458	300	3	10
7.				Dera stream	De	N47.65298	E18.97206	166	2	5
8.		MiddleDanubian	III. Lake Balaton	River Zala	Za	N46.85031	E16.62710	180	6	4
9.				Marótvölgyi canal	Mv	N46.50994	E17.29116	126	9	5
10.				Tapolca stream	Ta	N46.84992	E17.42141	124	10	8
11.				Egervíz stream	Eg	N46.83625	E17.47877	121	10	10
12.				Tetves stream	Te	N46.72650	E17.77647	127	10	8
13.			IV. RiverKapos-Sárvíz system	Séd stream	Se	N47.09030	E18.07058	140	10	10
14.				Baranya canal	Ba	N46.19167	E18.15551	144	3	7
15.				Völgységi stream	Vo	N46.28232	E18.47123	129	2	7
16.		SouthDanubian	V. River Dráva	Bükkösdi stream	Bu	N46.08520	E17.98406	124	9	10
17.				River Kerka	Kr	N46.81240	E16.34637	223	9	9
18.	Tiszaian	MiddleTiszaian	VI. „upper flow”River Tisza	Bózsva stream	Bz	N48.46353	E21.51624	150	10	9
19.				Máriapócsi canal	Mp	N47.96533	E21.97856	124	10	9
20.			VII. River Sajó	Hódos stream	Hd	N48.17728	E20.25596	181	2	5
21.				Abodi stream	Ab	N48.38829	E20.77152	201	2	10
22.			VIII. Lake Tisza	Kácsi stream	Kc	N47.93180	E20.63465	148	9	9
23.				Ostorosi stream	Os	N47.85569	E20.44571	151	2	10
24.			IX. River Zagyva	Leleszi stream	Le	N48.05221	E20.17948	205	4	8
25.				Zsunyi stream	Zs	N47.94147	E19.58816	225	4	10
26.			X. River Körös	Konyári-Kálló stream	Ko	N47.47491	E21.96314	135	10	8
27.				Villongó-ér stream	Vi	N47.39352	E21.85245	116	4	9

### Fish Sampling

Fish samples were collected between 2009 and 2012 by electrofishing from 27 sites across five sub-catchments of the Danube River and five sub-tributaries of the Tisza River (Table 1, Fig. 1). Since individuals of the *Gobio* genus show notable phenotypic plasticity, we investigated only adult (>60 mm standard length) specimens characterised by *Gobio gobio*- like morphological features, such as dispersedly spotted dorsal and caudal fins, and with no epithelial crests on the scales situated on the predorsal region of the body [35].

### Molecular Methods

#### DNA extraction and purification

Fin clips of 241 specimens were sampled and stored in 96% ethanol at −20°C until DNA extraction. DNA was isolated with a DNeasy Blood and Tissue kit (Qiagen, Germany), using 10–20 mg of fin tissue as per the manufacturer’s instructions. Quality and quantity of the extracted DNA were verified using a NanoDrop 2000c Spectrophotometer (Thermo Scientific, USA).

#### Mitochondrial sequence data

DNA of 168 out of 241 individuals (111 from the Danube River and 57 from the Tisza River catchments) were used for the amplification of the mitochondrial control region (mtCR). The sequences of mtCR were amplified by polymerase chain reaction (PCR) using the primers CR159 (CCCAAAGCAAGTACTAACGTC) and CR851 (TGCGATGGCTAACTCATAC) ([33]). PCRs were carried out using 0.2 µl of 5 U/µl Taq DNA polymerase (Fermentas), 2.5 µl of 10X Taq buffer, 1.7 µl MgCl_2_ (25 mM), 0.2 µl dNTPs (10 mM), 0.3 µl of each primer (20 µM), 2.0 µl template DNA, and 17.8 µl purified and distilled water in a final volume of 25 µl. Reactions were performed in a MJ Research PTC-200 Peltier Thermal Cycler under the following conditions: 95°C for 1 min, followed by 37 cycles of 94°C for 45 s, annealing at 52°C for 30 s, and an extension temperature of 72°C for 45 s, followed by a final extension at 72°C for 8 min. PCR products were purified from 1% agarose gel using the Millipore Ultrafree-DA DNA extraction kit. PCR products were sequenced on an ABI 3730XL sequencing machine by MWG-Biotech AG (http://www.mwg-biotech.com). Sequences were edited manually and aligned using the program Geneious 5.4 [43] and ClustalX 2.0.11 [44]. Newly described haplotypes have been deposited in the GeneBank. Calculation of sequence polymorphism and haplotype detachment was performed using DnaSP 5.10 software [45]. Sequence divergence was calculated with net nucleotide divergence (Da) in MEGA5 [46].

#### AFLP

To verify the results of the mitochondrial CR sequencing, a complementary method, Amplified Fragment Length Polymorphism (AFLP), was carried out; which is a reproducible, PCR-based molecular genetic method [41]. Altogether, 241 specimens were surveyed according to the following protocol. 200 ng DNA extracted from caudal fin tissue was digested at 37°C for 2 hours in a final volume of 10 µL with 2.5 U MseI, 5 U EcoRI enzymes and 2 µL NEBuffer4 (New England BioLabs, USA). Enzymes were then inactivated at 65°C for 20 min. Adaptor ligation was carried out at 24°C for 16 hours in 20 µL final volume containing the total digestion mixture, 0.25 µM EcoRI, 2.5 µM MseI adaptors, 200 cohesive end unit T4 Ligase and 1 × T4 Ligase Reaction Buffer (New England BioLabs). After heat inactivation at 65°C for 10 min, 10 µL of digested, ligated mixture was diluted 10 fold with nuclease free water. Pre-selective PCR was carried out with AmpliTaqGold 360 Master Mix (Applied Biosystems, USA) in a final volume of 20 µL containing 0.5 µM Eco-A (5′ GACTGCGTACCAATTCA 3′), 0.5 µM Mse-C primer (5′ GATGAGTCCTGAGTAAC 3′) and 5 µL diluted, digested, ligated DNA. The PCR was started at 94°C for 2 min followed by 20 cycles of 94°C for 30 sec, 56°C for 1 min, 72°C for 1 min and a final elongation at 72°C for 7 min. Selective PCR was performed in a final volume of 20 µL containing AmpliTaqGold 360 Master Mix, 0.1 µM fluorescently labelled Eco-ACT primer (5′ 6FAM GACTGCGTACCAATTCACT 3′), 0.25 µM Mse-CTT primer (5′ GATGAGTCCTGAGTAACTT 3′), 2 µL PCR product from the pre-selective PCR. Cycling conditions of the touchdown PCR were as follows: enzyme activation at 94°C for 2 min followed by 13 cycles for 30 sec at 94°C, for 30 sec at 65°C and decreased by 0.7°C in each cycle, and for 1 min at 72°C, then 23 cycles for 30 sec at 94°C, for 30 sec at 56°C and for 1 min at 72°C, followed by 5 min at 72°C. Digestion, ligation and PCRs were carried out in a Gene Amp PCR System 9700 (Applied Biosystems). Fragment analysis was performed on an ABI 3130 sequencer (Applied Biosystems, USA) and data were analysed with Peakscanner v1.0 (Applied Biosystems). Electropherograms were automatically analysed with tinyFLP [47], by the following scoring parameters: min. height: 90, max. width: 1, min. size: 50, max. size: 500, range (+/−): 0.5, min peak-peak dist.: 1, peak height difference: 0, min. freq.: 0.1, max. freq.: 90. From the 553 peaks detected in total, 154 selected bands were retained. After further evaluation (e.g. specimens with a small number of peaks were excluded from the analysis) a dataset of 229 specimens was used for further statistical analyses.

### Data Analysis

#### Mitochondrial sequence data

To shed light on the taxonomic relationships, alignment of all haplotypes found in this study and the previously published *Gobio* haplotypes (source: [31]) described from the neighbouring regions (e.g. Central Europe, Balkan Peninsula, and Anatolia) was performed (Table 2). Originally the sequences revealed in this study were 651 bp long, but for the Network analyses we had to align them to the GenBank sequences of these closely relative *Gobio* species. Therefore for the Network analyses a 652 bp long dataset were used. Network was constructed using the median-joining algorithm in Network v. 4.6. [48]. Similar haplotypes were classified arbitrarily into haplogroups (see “boxes” in Fig. 2). Differentiation within and among haplogroups was tested by analysis of molecular variance (AMOVA; [49]) with 9999 permutations.

**Table 2:** GenBank haplotypes used for the network computation. Numbers (N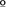) of haplotypes correspond with the numbers displayed in Figure 2.

		GenBank accession numbers				
code	taxon name by GenBank	N 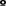 1	N 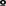 2	N 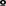 3	N 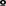 4	N 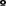 5
**‘A’**	*Gobio obtusirostris*	EU131554	EU131557	EU131558		
**‘C’**	*Gobio sp1#*	EU131564	EU131565	EU131563		
**‘D’**	*Gobio gobio*	EU131542	EU131544	EU131543	EU131545	EU131546
**‘E’**	*Gobio skadarensis*	EU131568	EU131569	EU131567		
**‘F’**	*Gobio carpathicus*	EU131561	EU131552	EU131560	EU131559	
**‘G’**	*Gobio ohridanus*	EU131572	EU131571	EU131573	EU131570	
**‘H’**	*Gobio insuyanus*	EU131576	EU131574	EU131578	EU131580	EU131579

(#: taxon name described in Mendel *et al.,* 2008).

To construct the phylogenetic tree, the (652 bp long) sequence set analyzed in the Network analysis was aligned against further haplotypes used as outgroup taxa of varying putative phylogenetic depths (sources: [50], [51], [31], and Mendel et al. unpublished data): *Romanogobio vladykovi* (GenBank a.n.: EF427385), *Romanogobio banaticus* (GenBank a.n.: EF427393), *Sarcocheilichthys variegatus* (GenBank a.n.: NC004694), *Rhodeus ocellatus kurumeus* (GenBank a.n.: AB070205). Thus the lenght of aligned sequence set was 666 bp. Bayesian phylogenetic analysis was conducted by Markov chain Monte Carlo method (B/MCMC), and it was performed in MrBayes 3.2 [52]. The best-fitting models of DNA substitution were selected for analysis using Akaike information criterion (AIC) implemented in the jModelTest 0.1.1 [53], [54]. jModelTest indicated that Hasegawa, Kishino and Yano substitution model [55] with gamma-distributed rate heterogeneity (α = 0.5710) (HKY+G) was the best fitting. We conducted Bayesian tree construction with 6 chains, 2 independent runs and 7 million generations. Trees were sampled every 1000th generation. The first 10000 generations were discarded as burn-in. We plotted the log-likelihood scores of sample points against generation time using Tracer 1.5 [56] to ensure that stationariness was achieved after the first 10000 generations by checking whether the log-likelihood values of the sample points reached a stable equilibrium plateau. We used the remaining trees with average branch lengths to create a 50% majority-rule consensus tree with the sumt option of MrBayes. Posterior probabilities were obtained for each clade.

#### AFLP analysis

Higher level differentiation of *Gobio* assemblages was assessed using STRUCTURE 2.3.3 [57] to estimate the most probable number of genetic groups (clusters, K) for all analysed individuals. Values of K were investigated from 1 to 10, with a burn-in period of 10000 followed by 100,000 iterations and 10 runs for each K using an admixture model with correlated allele frequencies. Results of the Bayesian statistics were evaluated by Structure Harvester [58] implementing the Evanno method [59]. To characterise the standard measures of population genetic diversity, the percentage of polymorphic loci (%), mean unbiased heterozygosity, and unbiased Nei's gene diversity [60] were calculated. Within population genetic distance (GD) was calculated using the following equation:
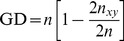
where 2n_xy_ equals the number of shared character states and n is the total number of binary characters. Population genetic structure was characterised by hierarchical AMOVA [49] with 9,999 randomisations. Isolation by distance was estimated by a Mantel test [61] using pairwise Φpt (similar to pairwise F_st_, [62] data). Nei unbiased genetic distances and pairwise hydrological distances were derived from a hydrological map (1∶10 000) with 9,999 randomisations. All of these calculations were made in GenAlEx v6.5 [63] statistical software. The inbreeding coefficient (F_is_) and fixation index, as a measure of population differentiation (F_st_) from AFLP markers, were computed using the Bayesian ABC4F software [64]. The percentage of polymorphic loci, mean unbiased heterzygosity, F_is_ and F_st_ values of the studied populations (where N≥8) were compared with the altitude of the sampling sites by Spearman rank correlations. We calculated the membership probabilities of each individual for the different a priori groups (i.e. populations, where N≥8 and geographical regions) based on retained discriminant functions using cross-validation with Discriminant Analysis of Principal Components (DAPC) [65]. All the files used for statistical analyses are available in the supplementary material.

## Results

### Mitochondrial DNA Sequence Analysis

Aligned sequences of 651 3′- end CR mtDNA were obtained from 168 individuals grouped into 17 haplotypes. Sequence data of 15 previously undescribed haplotypes are highlighted in bold in Table 3. These have been deposited in the GenBank database under Accession Nos. KC757328-42. The sequences of the CR03 and CR14 haplotypes had already been identified from the Danube catchment in the Czech Republic and in Slovakia [33], and were demonstrated by megaBLAST [66] to be *Gobio gobio* (100% similarity with the specimen: EU131542) and *G. carpathicus* (100% similarity with the specimen: EU131559) respectively. From the North Danubian region only three and from the Tisza River catchment a total of five haplotypes were displayed. The Middle and South Danubian regions were the richest in haplotypes. Moreover nine out of the 10 haplotypes found were unique to these regions (Table 3). Results of the median-joining Network analysis showed that haplotypes described from Hungary were classified into five haplogroups (A, B, C, D, F ‘boxes’ in Fig. 2). Through the AMOVA analysis, 85% of the total genetic variance was explained by among haplogroup differences, and in addition significant (p<0.001) differentiation was found in each pairwise comparison of haplogroups, confirming the arbitrary classification pattern.

**Table 3:** Details of sample locations, population code and the number of individuals (N) analysed by the two methods (mtCR, AFLP).

				Haplotype frequencies																					
Region	Sub-Catchmentarea	Pop.code	mtCR(N)	CR01	CR02	CR03	CR04	CR05	CR06	CR07	CR08	CR09	CR10	CR11	CR12	CR13	CR14	CR15	CR16	CR17	AFLP(N)	P.loci %	UHe	F_is_	F_st_
N. Danubian	I. R. Rába	Sz	2	**2**																	10	53.25	0.186	0.360	0.020
		Pi	3	**3**																	9	53.90	0.195	0.480	0.030
		Ge	3	**3**																	10	59.74	0.192	0.250	0.060
		Re	10	**10**																	10	63.64	0.202	0.410	0.010
	II. D.I. R. Danube	Cu	10	**6**	**2**	2															10	52.60	0.190	0.570	0.050
		Km	3	**3**																	10	62.34	0.206	0.140	0.110
		De	2	**2**																	5				
M. Danubian	III. L. Balaton	Za	6	**1**			**5**														4				
		Mv	9					**3**	**4**	**2**											5				
		Ta	10				**9**		**1**												8	58.05	0.162	0.320	0.050
		Eg	10	**8**			**2**														10	66.23	0.208	0.230	0.020
		Te	10	**2**					**8**												8	42.21	0.130	0.850	0.180
	IV. R. Kapos-Sárvíz	Se	10	**3**			**1**		**6**												10	61.04	0.201	0.370	0.030
		Ba	3						**3**												7				
		Vo	2	**1**					**1**												7				
S. Danubian	V. R. Dráva	Bu	9						**8**		**1**										10	50.65	0.183	0.420	0.090
		Kr	9						**3**			**2**	**1**	**1**	**2**						9	55.19	0.196	0.270	0.060
M. Tiszanian	VI. U.R. Tisza	Bz	10													**8**	2				9	52.60	0.186	0.710	0.070
		Mp	10													**7**		**1**	**2**		9	57.14	0.185	0.530	0.030
	VII. R. Sajó	Hd	2													**2**					5				
		Ab	2																**2**		10	59.74	0.222	0.270	0.100
	VIII. Lake Tisza	Kc	9													**2**			**7**		9	59.74	0.210	0.090	0.040
		Os	2																**2**		10	63.64	0.225	0.370	0.080
	IX. R. Zagyva	Le	4																**4**		8	48.70	0.175	0.480	0.080
		Zs	4																**4**		10	62.99	0.211	0.770	0.080
	X. R. Körös	Ko	10													**3**				**7**	8	63.90	0.194	0.460	0.030
		Vi	4													**4**					9	60.39	0.181	0.400	0.040
		∑	168	44	2	2	17	3	34	2	1	2	1	1	2	26	2	1	21	7	229	*52.48±11.2	*0.18±0.03	*0.48±0.24	*0.07±0.06
		%	100	26.2	1.2	1.2	10.1	1.8	20.2	1.2	0.6	1.2	0.6	0.6	1.2	15.5	1.2	0.6	12.5	4.2					

Haplotypes first described in this study are highlighted with bold. S.D.: South Transdanubian Region; (N): number of individuals, P.loci%: percentage of polymorphic loci; UHe: mean unbiased heterozygosity of a certain population; F_is_: Inbreeding coefficient of a certain population; F_st_: measure of population differentiation; *: mean±SD.

**Figure 3:**
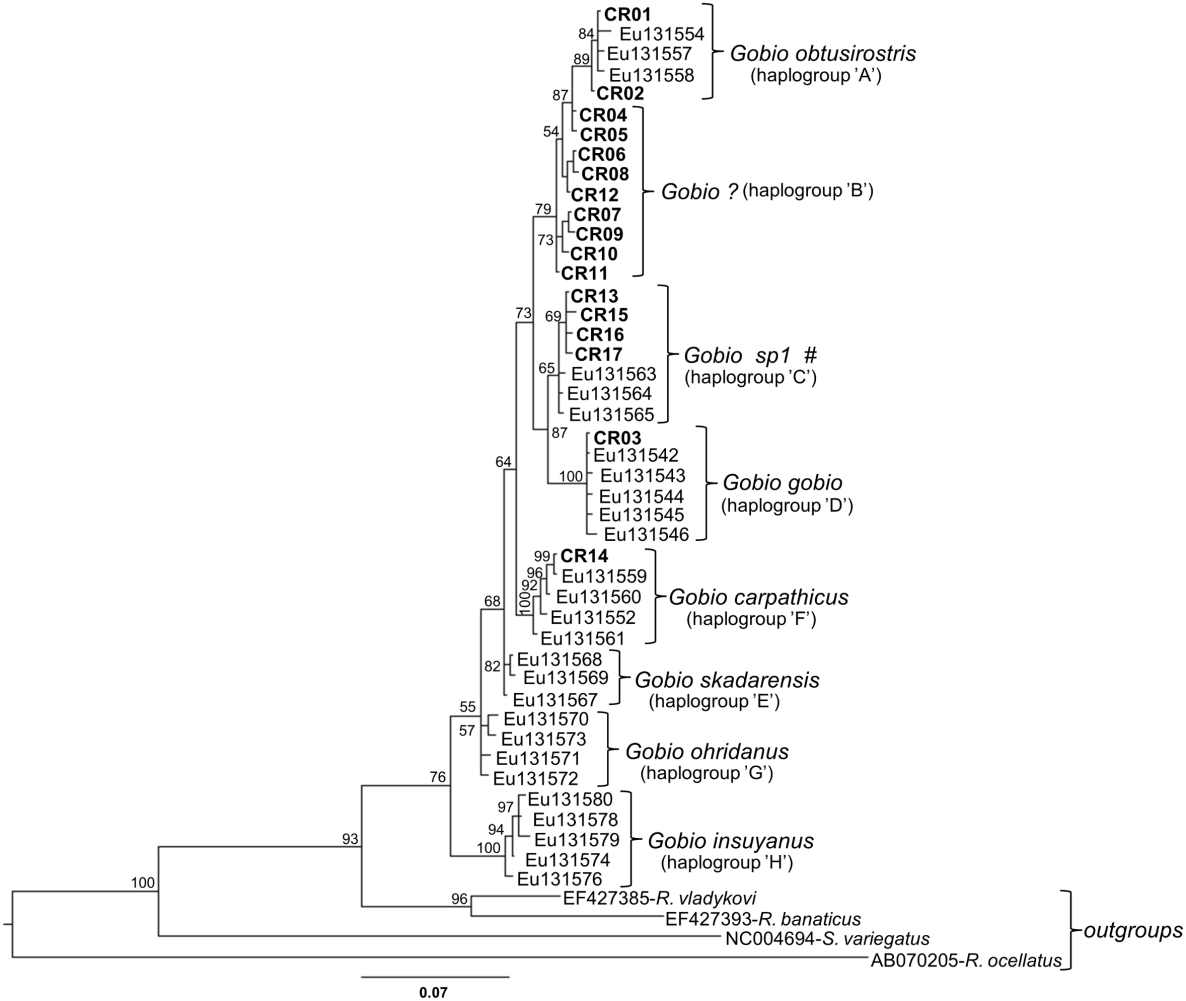
Bayesian consensus tree derived from the analysis of the mtCR sequence data. Haplotypes revealed in this study are marked with their CR codes (see Table 3). Bayesian posterior probabilities are assigned on nodes. For GenBank accession numbers see the text and Table 2. #: taxon name described in Mendel et al. (2008).

With the exception of haplogroup ‘B’, each one corresponds to a previously described “species” (Table 4). In the Network analysis, 46 investigated specimens were identified as *G. obtusirostris* (haplogroup ‘A’), 55 as *G. sp1* described by Mendel et al. [31] (haplogroup ‘C’), two as *G. gobio* (haplogroup ‘D’) and two as *G. carpathicus* (haplogroup ‘F’) (Fig. 2, Table 4). Altogether 63 specimens originating from the Middle and South Danubian region form a distinct, currently unidentified group (haplogroup ‘B’), which was in a transitional position between *G. sp1* and *G. obtusirostris* (Fig. 2). The results of Bayesian phylogenetic analysis (Fig. 3) were similar to those obtained through the Network analysis. However, the posterior probabilities of nodes showed a high level of uncertainly (weakly supported branching) at both “lower” (i.e. species) and at “higher” (i.e. genus) levels as well.

**Table 4:** Estimates of evolutionary divergence over sequence pairs within and between haplogroups (see Fig. 4) based on 652b long mtCR sequence data.

Haplogroup			‘A’	‘B’	‘C’	‘D’	‘E’	‘F’	‘G’	‘H’
	N	Taxonname by GenBank	*Gobio* *obtusirostris*	*Gobio ?*	*Gobio sp1#*	*Gobio* *gobio*	*Gobio* *skadarensis*	*Gobio* *carpathicus*	*Gobio* *ohridanus*	*Gobio* *insuyanus*
**‘A’**	5	*Gobio obtusirostris*	**0.37**±**0.23**	0.693**	0.743**	0.870**	0.830*	0.768**	0.825**	0.913**
**‘B’**	9	*Gobio ?*	1.31±0.31	**0.40**±**0.18**	0.732**	0.862**	0.832**	0.795**	0.859**	0.928**
**‘C’**	7	*Gobio sp1#*	1.98±0.18	1.61±0.17	**0.34**±**0.15**	0.796**	0.744**	0.739**	0.816**	0.898**
**‘D’**	6	*Gobio gobio*	3.35±0.26	2.65±0.17	1.99±0.21	**0.26**±**0.14**	0.852*	0.863**	0.877**	0.927**
**‘E’**	3	*Gobio skadarensis*	2.45±0.28	1.91±0.18	1.50±0.21	2.06±0.14	**0.20**±**0.09**	0.737*	0.749*	0.920*
**‘F’**	5	*Gobio carpathicus*	1.97±0.14	1.76±0.15	1.74±0.14	2.80±0.14	1.29±0.18	**0.31**±**0.17**	0.808**	0.913**
**‘G’**	4	*Gobio ohridanus*	2.80±0.28	2.77±0.21	2.71±0.21	3.27±0.14	1.38±0.17	2.24±0.17	**0.51**±**0.08**	0.877**
**‘H’**	5	*Gobio insuyanus*	5.09±0.31	5.09±0.21	4.35±0.26	4.86±0.20	3.87±0.23	4.42±0.21	3.48±0.21	**0.40**±**0.20**

N: the number of haplotypes in each box. Diagonal (bold): within haplogroup percentage genetic differences (mean±SD); above: Φpt values of pairwise AMOVA (* and ** indicate p<0.05 and p<0.01, respectively); below: between haplogroups percentage differences (mean±SD); (#: taxon name described in Mendel *et al.,* 2008).

#### AFLP

**Figure 4:**
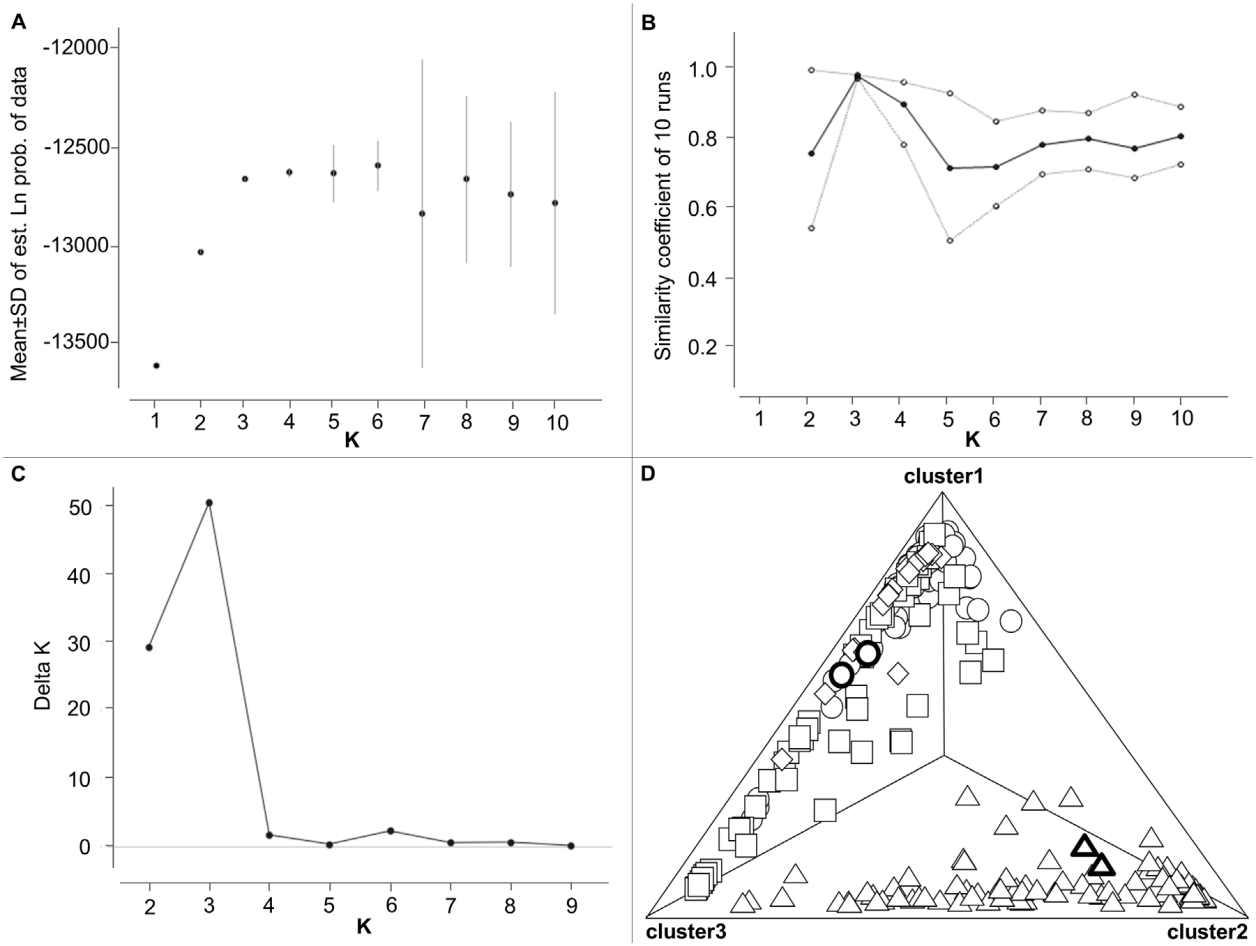
Determination of the number of clusters best fitting the AFLP data: STRUCTURE-based mean±SD likelihood values of ten runs for each K from K = 1 to 10 (A), similarity coefficient (min., mean and max values) of ten runs for each K from K = 1 to 10 (mean±SD) (B), Delta K statistic (C), and Triangle plot with allocation of individuals to clusters mapped according to K = 3 (D). Where ○: specimens from the North Danubian region (the two emphasized individuals are identified as *Gobio gobio* in the CR analysis), □: Middle Danubian sites, ⋄: South Danubian sites, ▵: specimens from the Tisza region (the two emphasized individuals are identified as *Gobio carpathicus* in the CR analysis).

The mean of the estimated Ln probability values from STRUCTURE analysis of the final matrix strongly increased between K = 1 and K = 3 and then consolidated at higher K values (Fig. 4a). The comparative statistics [67], [68] supported three major clusters (Fig. 4b, c). A triangle plot of the results (Fig. 4d) showed that individuals classified into the Cluster 1 originated only from the Danubian region while the individuals in Cluster 2 originated completely from the Tiszanian catchment area. The Cluster 3, consisting of mainly Middle and South Danubian specimens, shows a transitional position (continuous transition) between the two aforementioned clusters. This pattern was similar to the hydrological distances between the sampling sites (see Fig. 1c).

For population genetic analyses, data were used of those 21 populations (196 individuals) where N≥8. The average number of bands per specimen was 40.5±9.4 (ranging between 16.0 and 62.0). Base population genetic features as: P. loci %, UHe, F_is_, F_st_ are given for each population where N≥8 in Table 3. Kruskall-Wallis tests revealed that the North, Middle and South Danubian and Tiszanian groups of populations did not show any significant differentiation in terms of their population genetic features (Table 3).

**Figure 5:**
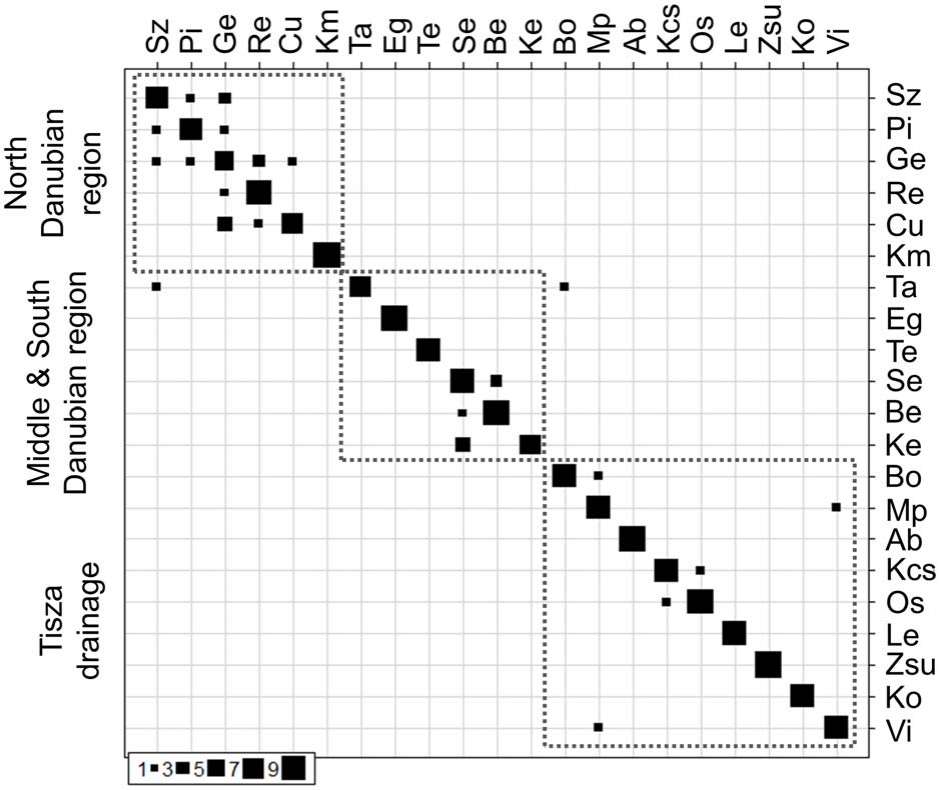
Plots of cross-validation tables for AFLP data based on DAPC. Correctly classified individuals are placed on the diagonal. The square size equals the number of individuals of posterior group assignment based on posterior probabilities. Rows correspond to actual sites (a priori), while columns correspond to inferred sites (posteriori). Squares with broken lines show regional detachments of populations.

Within population genetic distance (GD), pairwise Φpt and pairwise Unbiased Nei Genetic Distances data are displayed in Table 5. GD ranged between 25.4 and 38.8 (av. ±SD = 34.8±3.1) and neither differed significantly by subregion, nor correlated with the altitudinal position of the collection site. Mean Unbiased Nei Genetic Distances ranged between 0.007 and 0.100 (av. ±SD = 0.035±0.020). AMOVA analysis showed that among group differences accounted for 12% of the total genetic variance, and 193 out of the 210 pairwise comparisons (93%) showed significant (p<0.05) differentiation. Pairwise Φpt data ranged between zero and 0.321 (av. ±SD = 0.116±0.06). This markedly strong population separation was verified by the results of DAPC. Assessing the AFLP dataset, 86% and 99% of the individuals were grouped correctly on population and region levels respectively in multidimensional space based on the cross-validation procedure within DAPC (Fig. 5). Results of Spearman rank correlations supported that the population genetic variables (P. loci %, UHe, F_is_, and F_st_) were not significantly correlated (p<0.05) with the altitudinal position of the sites.

**Table 5:** Population level differences of the studied *Gobio* populations based on the AFLP. Data are only displayed for those 21 populations where N≥8.

N 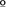		1.	2.	3.	4.	5.	6.	10.	11.	12.	13.	16.	17.	18.	19.	21.	22.	23.	24.	25.	26.	27.
	Code	Sz	Pi	Ge	Re	Cu	Km	Ta	Eg	Te	Se	Bu	Kr	Bo	Mp	Ab	Kcs	Os	Le	Zsu	Ko	Vi
1.	Sz	**32.2**±**5.0**	0.012	0.006	0.004	0.026	0.083**	0.089**	0.076**	0.160**	0.016	0.076**	0.071**	0.060**	0.128**	0.152**	0.196**	0.150**	0.198**	0.140**	0.175**	0.128**
2.	Pi	0.012	**35.1**±**5.5**	0.040*	0.045**	0.024	0.053**	0.076**	0.077**	0.177**	0.035*	0.083**	0.063**	0.089**	0.113**	0.155**	0.167**	0.138**	0.205**	0.136**	0.181**	0.127**
3.	Ge	0.010	0.024	**36.0**±**6.0**	0.017	0.000	0.056**	0.105**	0.095**	0.177**	0.033*	0.076**	0.074**	0.082**	0.097**	0.136**	0.188**	0.127**	0.157**	0.129**	0.152**	0.125**
4.	Re	0.012	0.016	0.014	**37.3**±**5.9**	0.028	0.064**	0.104**	0.101**	0.173**	0.051	0.076**	0.091**	0.094**	0.114**	0.172**	0.188**	0.152**	0.177**	0.163**	0.172**	0.136**
5.	Cu	0.012	0.011	0.007	0.007	**33.9**±**4.8**	0.063**	0.066**	0.081**	0.143**	0.047	0.082**	0.069**	0.055*	0.095**	0.158**	0.170**	0.116**	0.161**	0.120**	0.145**	0.090**
6.	Km	0.032	0.026	0.034	0.026	0.027	**36.5**±**5.4**	0.148**	0.095**	0.229**	0.064	0.099**	0.051**	0.126**	0.137**	0.141**	0.137**	0.128**	0.177**	0.136**	0.201**	0.179**
10.	Ta	0.021	0.026	0.032	0.028	0.018	0.049	**30.5**±**5.1**	0.068**	0.044*	0.038*	0.075**	0.099**	0.103**	0.082**	0.221**	0.244**	0.194**	0.196**	0.204**	0.116**	0.052*
11.	Eg	0.027	0.027	0.033	0.028	0.022	0.036	0.027	**37.1**±**4.4**	0.156**	0.031*	0.071**	0.073**	0.081**	0.085**	0.140**	0.156**	0.120**	0.177**	0.150**	0.126**	0.087**
12.	Te	0.027	0.037	0.036	0.036	0.024	0.061	0.011	0.035	**25.4**±**6.4**	0.095	0.123**	0.185**	0.149**	0.122**	0.290**	0.321**	0.259**	0.233**	0.253**	0.192**	0.101**
13.	Se	0.015	0.021	0.020	0.023	0.016	0.032	0.016	0.021	0.021	**36.7**±**4.9**	0.033	0.032*	0.069**	0.074**	0.130**	0.168**	0.132**	0.156**	0.133**	0.130**	0.087**
16.	Bu	0.022	0.022	0.020	0.016	0.015	0.029	0.017	0.021	0.022	0.015	**31.4**±**6.8**	0.045*	0.105**	0.120**	0.192**	0.230**	0.183**	0.225**	0.192**	0.194**	0.156**
17.	Kr	0.024	0.025	0.032	0.030	0.024	0.033	0.028	0.032	0.044	0.019	0.019	**33.0**±**5.1**	0.113**	0.131**	0.159**	0.185**	0.144**	0.209**	0.157**	0.201**	0.171**
18.	Bo	0.020	0.028	0.027	0.024	0.014	0.041	0.030	0.021	0.031	0.022	0.022	0.038	**34.2**±**5.8**	0.012	0.063**	0.092**	0.047**	0.096**	0.023	0.109**	0.079**
19.	Mp	0.036	0.037	0.033	0.027	0.022	0.045	0.025	0.028	0.030	0.021	0.030	0.043	0.016	**36.9**±**6.1**	0.089**	0.122**	0.061**	0.050*	0.076**	0.080**	0.054**
21.	Ab	0.045	0.056	0.047	0.054	0.050	0.045	0.065	0.044	0.071	0.035	0.053	0.050	0.030	0.029	**36.5**±**4.1**	0.033**	0.000	0.118**	0.019	0.158**	0.167*
22.	Kcs	0.067	0.071	0.066	0.065	0.059	0.059	0.083	0.059	0.100	0.063	0.070	0.070	0.041	0.051	0.031	**36.1**±**4.7**	0.029	0.117**	0.032**	0.183**	0.176**
23.	Os	0.038	0.044	0.035	0.039	0.029	0.043	0.048	0.036	0.055	0.033	0.041	0.045	0.018	0.019	0.009	0.025	**37.7**±**5.7**	0.065**	0.019	0.123**	0.132**
24.	Le	0.047	0.054	0.042	0.045	0.037	0.053	0.044	0.043	0.046	0.034	0.047	0.058	0.030	0.019	0.033	0.046	0.020	**30.8**±**6.9**	0.091**	0.141**	0.130**
25.	Zsu	0.039	0.041	0.042	0.047	0.033	0.045	0.055	0.041	0.055	0.036	0.045	0.051	0.018	0.026	0.018	0.030	0.016	0.029	**37.1**±**4.5**	0.144**	0.133**
26.	Ko	0.042	0.050	0.045	0.045	0.035	0.067	0.031	0.038	0.039	0.039	0.046	0.058	0.030	0.026	0.050	0.068	0.030	0.036	0.041	**34.6**±**6.1**	0.025
27.	Vi	0.032	0.036	0.038	0.033	0.020	0.060	0.016	0.030	0.017	0.026	0.034	0.047	0.024	0.016	0.052	0.067	0.033	0.033	0.038	0.013	**36.7**±**5.9**

Diagonal: within population genetic distances (GD); above: pairwise Φpt calculated by AMOVA (* and ** indicate p<0.05 and p<0.01 respectively); below: pairwise Unbiased Nei Genetic Distances.

According to the results of Mantel tests, hydrological distances correlated significantly and positively both with Unbiased Nei genetic distances (Rxy = 0.499, p<0.01) and with the pairwise Φpt values (Rxy = 0.551, p<0.01) of the selected (N≥8) populations.

## Discussion

Our results only partially support the earlier assumptions of the taxonomic composition and distribution of *Gobio* species in the inner Carpathian Basin [31], [35], and show that *Gobio* reveal high diversity, both taxonomically and from the population genetic standpoint, within this relatively small and well-connected catchment area. At the same time, the presence of more cryptic species is indicated and the fine-scale separation of the identified genetic lineages between sub-catchments supports the existence of ecological barriers and on-going speciation in Hungarian drainage systems.

### Taxonomic and Phylogenetic Features

All of the *Gobio* haplotypes found in the inner Carpathian basin can be classified into the north European clade and all except one haplotype (*G. gobio*) belong to the north-eastern European subclade described by Mendel et al. [31]. At the same time, our results indicate that the taxonomic status of the stream-dwelling gudgeons inhabiting the inner area of the Carpathian Basin is more complex than was previously presumed. Although no remarkable differences were detected in the morphological and meristic traits of the specimens analyzed, haplotypes of three previously described species *(G. obtusirostris, G. gobio,* and *G. carpathicus*), a doubtful taxon (*G. sp1*) and an additional, distinct haplogroup were distinguished from the study area.

Although the haplotype of *G. carpathicus* occurred in the Tisza River catchment, this area was dominated by the haplotypes of *G. sp1* (Fig. 2, Table 4). Gudgeon stocks inhabiting the Danubian part of the country showed greater taxonomic complexity. Contrary to the earlier hypotheses [35], we found the haplotype of *G. gobio* in the Carpathian Basin (in Cuhai-Bakony-ér). Furthermore, haplotypes of *G. obtusirostris* proved to be dominant only in the North Danubian region. A distinct, but highly diverse haplogroup (haplogroup ‘B’) was dominant in the Middle and South Danubian regions (Fig. 2, Table 4).

**Figure 6:**
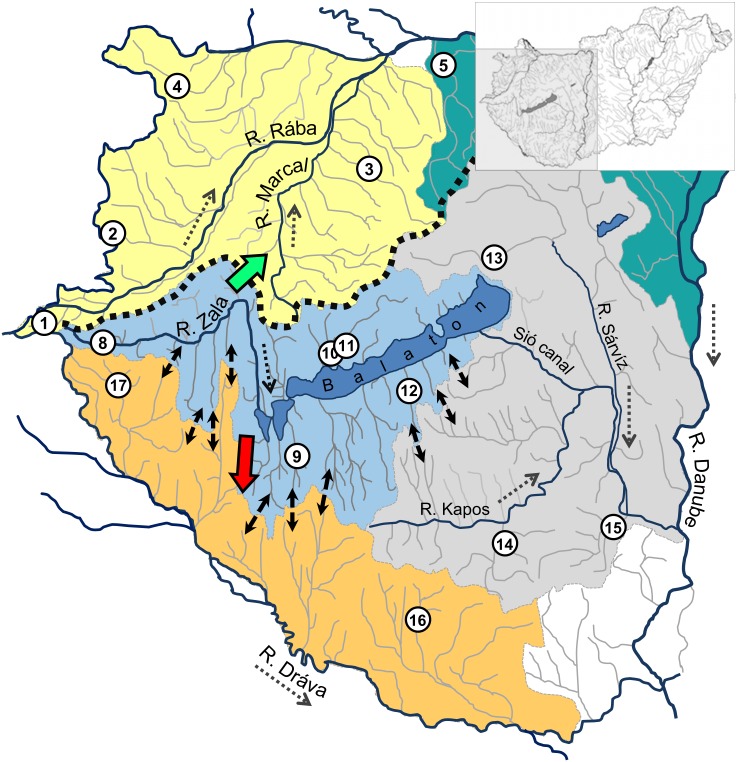
Recent river network of the Danubian region (Hungary). The four subcatchment areas are indicated by different colours. Flow direction of Zala River during the Middle and Late Pleistocene is indicated by blackframed green and red arrows respectively. The thick broken line indicates the Pleistocene watershed between the North and Middle Danubian regions. Intra-valley drainage divides (Síkhegyi, 2002) are shown by black bidirectional arrows. Numbered circles: sampling sites displayed in Table 1. Dotted arrows: recent flow direction.

Differentiation of haplogroups ‘A’ and ‘B’ (Table 4) may be attributed to a population split caused by paleohydrographic changes that took place in the geologic recent past. Namely, approximately 140,000 years ago a new watershed with an east-west direction formed, separating the North and Middle Danubian region [69]. This changed the flow direction of the Zala River, originally flowing northward following the current channel of the Marcal River, southward to the Dráva River. Separation of the Middle and South Danubian regions began only at the end of the Pleistocene, 14–16,000 years ago by the formation of Lake Balaton [70], [71]. Namely, the Zala River, and all the smaller streams flowing southward until then, turned toward this newly formed depression. The phylogenetic effect of the relatively old watershed separating the North and Middle Danubian region and the incomplete splits - “intra-valley drainage divides”- (Fig. 6) between the Middle and the Southern regions [72] was proven by the fact that (1) the Network analysis showed significant differentiation between haplogroup ‘A’ and ‘B’, but (2) haplotypes did not differ between the drainages of Lake Balaton, River Kapos-Sárvíz-system, and River Dráva (Fig. 2, Table 4).

Although some specimens characterised by CR01 (*G. obtusirostris*) haplotypes also occurred in the Middle Danubian region, their presence was restricted to only those sites that were in the vicinity of fish ponds and/or where trout (*Oncorhynchus* and/or *Salmo*) stocking occurred [73]. Therefore, we assume unintentional *G. obtusirostris* introductions with gamefish (trout) stocking and thus a secondary, anthropogenic contact between the phylogenetically separated haplogroups.

Haplogroup ‘B’ showed similar genetic distances from the *G. obtusirostris* (1.31%±0.31%) and from *G. sp1* (1.61%±0.17%), as an interspecific genetic divergence among some already accepted gudgeon species such as *G. skadarensis* and *G. carpathicus* (1.29%±0.18%) or *G. skadarensis* and *G. ohridanus* (1.38%±0.17%) (Table 4). These differences make species level detachment of the haplogroup ‘B’ or *G. sp1* taxon as well. At the same time, the results of Bayesian phylogenetic tree analysis showed weakly supported differentiation among haplogroup ‘B’ and some already accepted *Gobio* species (e.g. *G. obtusirostris*), and did not clearly support the recently accepted within-genus taxonomy. Our results indicate that the reproductive isolation of these entities may have only began in the geohistorical recent past, presumably in the middle Pleistocene. Contrary to the findings of the Network analysis, Bayesian phylogenetic computations in most cases query the species level differentiation within the *Gobio* genus. This premise is supported by the fact that in the case of other fish species, e.g. topmouth gudgeon - *Pseudorasbora parva* (Temminck & Schlegel, 1842), stone loach - *Barbatula barbatula* (Linnaeus, 1758), and grayling - *Thymallus thymallus* (Linnaeus, 1758), a similar or higher degree of differentiation among haplogroups is considered to be not more than subspecies level detachment [20], [74], [75]. In addition, some authors [76], [77] have suggested that the genetic distance of haplogroups must be greater than or equal to ten times the level of within-haplogroup differences to distinguish separate species. In our study, the *G. insuyanus* is the only taxon which fulfils the above mentioned criteria (Table 4).

### Population Genetic Variables

The values of basic population genetic parameters (P. loci%, UHe, F_is_ F_st_, and GD) did not show significant differences among subdrainage basins. Moreover, none of these variables showed significant correlation with the altitudinal position (as a possible marker for population isolation due to differences in the habitat use of fish) of the sampling sites. In the case of basic population genetic parameters, the local environmental conditions and the degree of hydrographic isolation are likely to be more important than either the altitudinal position or the taxonomic arrangement of the inhabiting specimens.

The population genetic features of gudgeon assemblages inhabiting the northwest region of Hungary differed notably from the other studied Hungarian assemblages. It is the only area where statistical analyses suggested considerable gene flow (Fig. 5, Table 5). This may be attributable to the species level differences. At the same time, landmarks of the river systems characterising this area assure convenient migration routes among sites. Similarly, the occurrence of *Gobio gobio* (haplotype) may be attributed to the role of Danube River. At the other Danubian Regions the population structure was much more differentiated. For the Middle Danubian Region, notable differences were found among closely situated sites. Results of the mtCR analyses revealed the existence of a different haplotype (CR01) in addition to the assumed “native” haplotype group from this area (Table 3). Therefore these differences may be caused by accidental *G. obtusirostris* introductions to this area.

Results of STRUCTURE analysis are in accordance with the results of mtCR sequence analyses. Both inferred the existence of three larger clusters/haplogroups within the Carpathian Basin. Furthermore, both analyses indicated the transitional position of Cluster 2/haplogroup ‘B’ between the Cluster 1/haplogroup ‘A’ and the Cluster 3/haplogroup ‘C’. The two specimens identified as *G. gobio* and two specimens identified as *G. carpathicus* by mtCR analyses did not show notable separation from the others by AFLP analysis (Fig. 4D) which may suggest interspecific hybridisation in these cases. There are numerous reports of interspecific and intergeneric (*Romanogobio* and *Gobio*) hybridisation [23], [78], [79] of European gudgeon species and our results support these findings.

Mantel test results revealed a clear pattern of isolation by hydrographical distance. Taxonomic and population genetic differences of the studied *Gobio* stocks were simultaneously changed by the growing hydrographical distances. This natural pattern is just slightly diminished by anthropogenic impacts.

Consequently, the genetic analyses confirmed the results of former analyses, which were based on mainly morphologic/morphometric variables [23], since they revealed that the Middle European *Gobio “*species” form an extremely diverse and variable group. At the same time explanation of the phylogenetic relationships and within-genus taxonomic features are still partly unresolved. Our results showed that because of the casual immigration and/or the accidental introductions, and the sympatric occurrences, the location of the collection site is not a convenient feature to discriminate these “species” occurring in Hungarian waters.

Our results indicate that these cryptic *Gobio* entities form a relatively young phylogenetic group and that the genetic differences among them are not strong enough in most cases for species level differentiation. Moreover, considering the possibilities of interspecific and intergeneric hybridisation, the recent taxonomic partitioning of the *Gobio* genus needs re-evaluation. However, the discovered genetic diversity is probably very vulnerable. Since the separation of haplogroups seems to be only an intermediate phase of an on-going speciation and stream-dwelling gudgeons have specific environmental needs and a restricted habitat area at present, habitat alteration and accidental stocking may easily damage the integrity of haplogroups. Consequently, conservation actions should be implemented to preserve the exceptional diversity of this fish group.
